# Multi-Biometric Feature Extraction from Multiple Pose Estimation Algorithms for Cross-View Gait Recognition

**DOI:** 10.3390/s24237669

**Published:** 2024-11-30

**Authors:** Ausrukona Ray, Md. Zasim Uddin, Kamrul Hasan, Zinat Rahman Melody, Prodip Kumar Sarker, Md Atiqur Rahman Ahad

**Affiliations:** 1 Department of Computer Science and Engineering, Begum Rokeya University, Rangpur 5404, Bangladesh; ausrukonaray@gmail.com (A.R.); kamrulhasanrony111@gmail.com (K.H.); prodip@brur.ac.bd (P.K.S.); 2 Department of Electrical and Electronic Engineering, Begum Rokeya University, Rangpur 5404, Bangladesh; zinatrahman0000@gmail.com; 3 Department of Computer Science and Digital Technologies, University of East London, London E16 2RD, UK

**Keywords:** gait recognition, skeleton-based gait recognition, human pose estimation algorithm, feature-level fusion, decision-level fusion, residual graph convolutional network

## Abstract

Gait recognition is a behavioral biometric technique that identifies individuals based on their unique walking patterns, enabling long-distance identification. Traditional gait recognition methods rely on appearance-based approaches that utilize background-subtracted silhouette sequences to extract gait features. While effective and easy to compute, these methods are susceptible to variations in clothing, carried objects, and illumination changes, compromising the extraction of discriminative features in real-world applications. In contrast, model-based approaches using skeletal key points offer robustness against these covariates. Advances in human pose estimation (HPE) algorithms using convolutional neural networks (CNNs) have facilitated the extraction of skeletal key points, addressing some challenges of model-based approaches. However, the performance of skeleton-based methods still lags behind that of appearance-based approaches. This paper aims to bridge this performance gap by introducing a multi-biometric framework that extracts features from multiple HPE algorithms for gait recognition, employing feature-level fusion (FLF) and decision-level fusion (DLF) by leveraging a single-source multi-sample technique. We utilized state-of-the-art HPE algorithms, OpenPose, AlphaPose, and HRNet, to generate diverse skeleton data samples from a single source video. Subsequently, we employed a residual graph convolutional network (ResGCN) to extract features from the generated skeleton data. In the FLF approach, the features extracted from ResGCN and applied to the skeleton data samples generated by multiple HPE algorithms are aggregated point-wise for gait recognition, while in the DLF approach, the decisions of ResGCN applied to each skeleton data sample are integrated using majority voting for the final recognition. Our proposed method demonstrated state-of-the-art skeleton-based cross-view gait recognition performance on a popular dataset, CASIA-B.

## 1. Introduction

Gait recognition is a popular behavioral biometric task that identifies individuals based on their unique walking patterns. Compared to physiological biometrics such as face, iris, DNA, and fingerprint recognition, gait recognition offers the advantage of long-distance identification and is difficult to disguise. Therefore, gait recognition holds significant potential for real-world applications, including forensic analysis, visual surveillance, and criminal investigation, particularly through security camera footage [1].

Existing gait recognition approaches [2,3,4,5,6,7,8] fall into two groups: appearance-based approaches, and model-based approaches. The appearance-based gait recognition approaches [2,3] use background-subtracted silhouette sequences to extract the gait features. Examples of the widely used template-based approach include gait energy image (GEI) [7], which is the average of a silhouette sequence over a gait cycle, gait flow image (GFI) [9], and chrono gait image (CGI) [10] techniques. Recently, silhouette sequences have also been directly employed to extract gait features: GaitSet [2], GaitPart [3], and GaitGL [4]. The appearance-based approaches have been more popular than model-based methods in the gait recognition community, due to their simplicity, easy-to-compute features, and high recognition accuracy. However, the limitations of the appearance-based approaches include drastic appearance changes in real-world applications due to variations in clothing, carried objects, camera view angle, imperfect background subtraction, sources of illumination, and cluttered backgrounds.

By contrast, model-based approaches [11,12,13,14,15,16] are less sensitive to variations in shape and appearance, focusing on analyzing gait by reconstructing a human kinematic model. This involves extracting features such as joint positions and angles, limb lengths, and the relative arrangement of body parts [17]. However, fitting an appropriate model is challenging, requiring high-resolution image sequences, and computationally expensive. Consequently, model-based approaches have been less explored in the literature. Several deep learning (DL)-based methods have recently been developed for human pose estimation (HPE) algorithms to extract human poses (i.e., skeletal key points) and can be categorized into top-down and bottom-up approaches. A top-down approach first detects the individual human bodies within an image and then estimates the pose for each detected individual; examples of top-down approaches include AlphaPose [15] and HRNet [16]. In contrast, a bottom-up approach starts by detecting body parts across the entire image, without initially separating individuals. The detected body parts are then grouped to form complete poses for each individual in the image; OpenPose [14] is a widely used method in this category.

**Figure 1:**
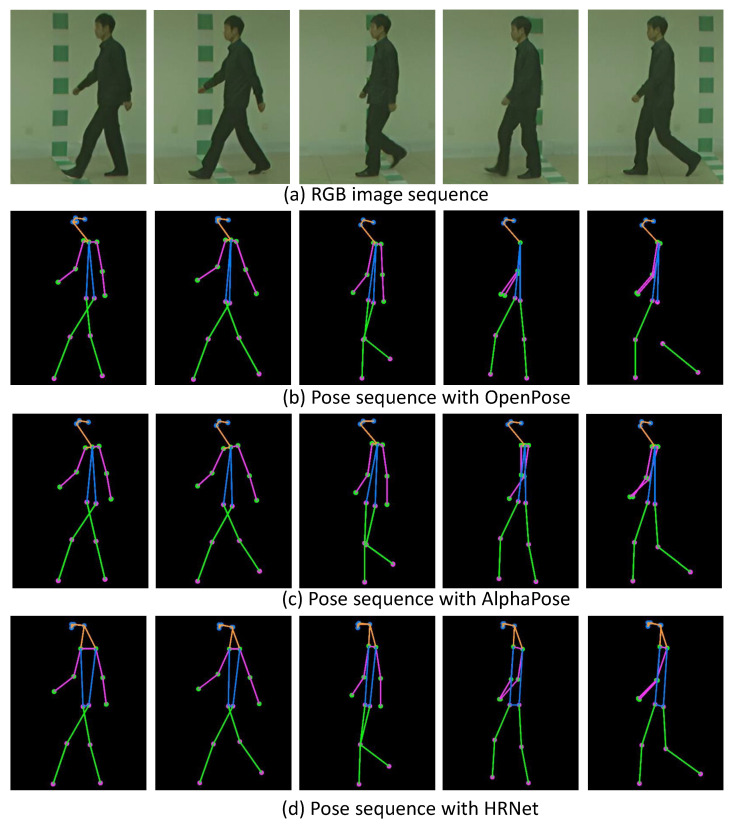
Example of a gait sequence for a subject (every fifth frame of a sequence): (**a**) RGB image sequence, (**b**) pose sequence with OpenPose, (**c**) pose sequence with AlphaPose, (**d**) pose sequence with HRNet. HRNet consistently generates accurate skeletons, while AlphaPose and OpenPose struggle with keypoint detection during self-occlusions, especially at the right shoulder in side views, with OpenPose occasionally losing body segments during leg swings.

Recently, several studies have leveraged the abovementioned HPE algorithms to obtain skeleton data points for gait recognition. For example, the studies in [11] employed a pre-trained model of OpenPose [14] to acquire human skeleton data points and to introduce a pose-based temporal-spatial network (PTSN) that takes a sequence of extracted skeleton data points to obtain features, demonstrating its performance on a publicly available gait dataset, CASIA-B [18]. Similarly, Teepe et al. [19,20] generated skeleton data points using HRNet [16] and proposed an approach called GaitGraph for gait recognition, which combines skeleton data points with graph convolutional networks (GCNs) [21], showing its effectiveness for cross-view gait recognition. Other recent studies [22,23,24] have utilized skeleton data to capture dynamic dependencies for improved gait recognition. These approaches used fixed single-pose estimation algorithms to generate skeleton pose data, which are then fed into DL models for feature extraction. However, each HPE algorithm presents unique strengths and weaknesses that impact the performance of DL-based approaches. For example, OpenPose detects body parts first and then assembles them into full poses, making it particularly efficient in multi-person scenarios but potentially less precise in complex occlusions. Conversely, AlphaPose detects individuals first, providing high accuracy and robustness against occlusions, clothing variations, and carried objects, though it can be slower in multi-person detection. HRNet maintains high-resolution representations throughout its network, leading to exceptionally precise keypoint detection and robustness across diverse conditions, but often at the cost of increased computational demands. Figure 1 illustrates skeleton data points generated by OpenPose, AlphaPose, and HRNet, highlighting their distinct characteristics in pose estimation.

To address the limitations of using a single biometric trait, researchers have explored multi-biometric techniques [25,26,27] based on fusion strategies to enhance identification accuracy and robustness. For example, the approach in [26] extracted multiple modality samples from a single source video, such as the face, gait, and height features, and employed score-level fusion for identification, while the approaches in [27,28] utilized multiple face samples. Additionally, several studies have investigated multi-algorithm systems [25] using a single biometric modality, where features are extracted using various algorithms and the final decision is made by combining these at the feature, score, or decision level. For example, Mehraj et al. [29] used AlexNet and VGG16 to extract features from face images, combining them at the feature level and feeding them into support vector machines (SVM) for final identification, while principal component analysis (PCA) and modular kernel PCA were employed in [30].

Inspired by the aforementioned multi-biometric techniques, this study proposes a robust framework that integrates multiple HPE algorithms, OpenPose, AlphaPose, and HRNet, to capture diverse and complementary skeleton data points, thereby enhancing gait recognition accuracy. By combining these state-of-the-art algorithms, our framework maximizes data diversity, a critical factor for reliable gait recognition across varying conditions. Specifically, we employ the ResGCN model [31] to extract comprehensive spatiotemporal features from skeleton data and apply both feature-level fusion (FLF) and decision-level fusion (DLF) techniques. These fusion approaches enhance the system’s robustness against common covariates such as clothing variations, carried objects, and varying camera angles. Our objective was to achieve state-of-the-art accuracy in skeleton-based gait recognition, which was validated through testing on the CASIA-B dataset, to demonstrate the framework’s efficacy. Finally, this study emphasizes the real-world potential of this multi-biometric approach for practical applications, particularly in forensic analysis and surveillance, demonstrating its ability to provide robust identification under diverse and challenging conditions. The primary contributions of this study are summarized as follows:

- We introduce a multi-biometric framework for gait recognition that leverages multiple human HPE algorithms, incorporating both top-down and bottom-up approaches. Specifically, we utilize state-of-the-art HPE algorithms: OpenPose, AlphaPose, and HRNet to generate comprehensive and diverse skeleton data points from a single video source. Following this, we employ ResGCN to extract rich spatiotemporal features from the generated skeleton data points.
- To enhance the robustness and accuracy of gait recognition, we apply both FLF and DLF in an ensemble manner. This approach effectively integrates the strengths of the features and decisions derived from ResGCN for each HPE-derived skeleton data point.
- We evaluated the proposed framework on a publicly available CASIA-B dataset. The results showed that it achieved state-of-the-art accuracy for skeleton-based cross-view gait recognition.

## 2. Related Work

### 2.1. Appearance-Based Approaches

Appearance-based gait recognition approaches analyze human walking patterns from RGB video sequences or background-subtracted silhouettes to identify an individual. These approaches can be categorized into template-based and sequence-based methods. In template-based approaches, a single gait template image is generated by aggregating spatiotemporal information of a person’s gait. For instance, Bhanu et al. [7] introduced a gait energy image (GEI) generated by averaging height-normalized silhouette sequences over a gait cycle. Various variations of GEI have since been proposed, including the gait entropy image (GEnI) [32], chrono gait image (CGI) [10], and gait flow image (GFI) [9] methods. The GEnI method uses computational entropy to encode dynamic features of a gait cycle into a single image, capturing robust temporal features under different covariate conditions. The GFI approach overlays an optical flow field onto a sequence of silhouettes, encoding the motion of a gait cycle into a single image. These template-based methods are easy to implement due to their model-free nature and simplicity. However, in real-world scenarios, various covariates, such as clothing variations, carried objects, and illumination changes, hinder the generation of an accurate template image, resulting in a lower recognition accuracy, as they fail to capture temporal motion information.

Recently, researchers have explored sequence-based approaches [2,3,4,33], where the entire gait silhouette sequence is used as input to extract spatiotemporal features. For example, Chao et al. [2] proposed a simple and effective model called Gaitset, which considered the silhouette sequence as a set. Initially, a sequence of 2D convolution neural networks (CNNs) and 2D max-pooling were used to obtain an intermediate feature map. Later, they used set pooling (SP) in the temporal direction to extract spatiotemporal features and employed horizontal pyramid mapping (HPM) to obtain stripe-based features. In addition, Fan et al. introduced GaitPart [3], where they used a frame-level part feature extractor (FPFE) to obtain part-based features, and employed a micromotion capture module (MCM) to capture motion features. Similarly, GaitGL [4] obtained part-based global and local features from a global and local feature extraction (GLFE) module using 3D CNNs. Later, generalized mean pooling (GeM) was employed to obtain the final feature representation. In addition, Chai et al. [33] introduced Lagrange gait, a method that utilizes the Lagrange equation to analyze the human walking process and determines that second-order information in the temporal dimension, which is crucial for gait recognition.

### 2.2. Model-Based Approaches

In the early era of model-based gait recognition, several methods were developed using manually modeled human body shape and motion during walking. In particular, features were extracted from key joints and various body parts, such as the position of the hips, knees, ankles, and feet. Recently, the model-based approach has used skeleton data points extracted using a Kinect sensor or HPE algorithms from RGB images. Recently, CNN-based HPE algorithms have been developed, such as OpenPose [14], AlphaPose [15], and HRNet [16], and these algorithms have brought a great change for studies on skeleton-based gait recognition. In contrast to silhouettes, skeleton motion proves to be more robust against external covariates [23], such as occlusions, variations in clothing, and objects being carried, due to its focus solely on the subject’s movements.

Several studies have explored DL-based methods for skeleton-based gait recognition. For example, the studies in [11] introduced a pose-based temporal-spatial network (PTSN) that takes a sequence of extracted skeleton data points to obtain features and demonstrated its performance on a publicly available gait dataset, CASIA-B [18]. Similarly, Liao et al. [12] proposed PoseGait, a method that employs 3D pose estimation coupled with handcrafted features. Utilizing 3D key points in Euclidean space, PoseGait calculates joint angles, joint motions, and bone lengths. These handcrafted features are then utilized by a CNN to extract high-level spatio-temporal features, while the approach in [34] extracts both dynamic and static features from human skeletons by employing techniques of disentangled learning. Moreover, Zhang et al. [35] introduced Gait-TR, a skeleton-based gait recognition framework, to integrate spatial transformer mechanisms with temporal convolutional networks for improved performance.

Graph convolutional networks (GCNs) have emerged as powerful tools in skeleton-based gait recognition, leveraging the relational structure of skeletal data to capture spatiotemporal dependencies between key joints. Teepe et al. [19] introduced GaitGraph, a model that integrates a GCN architecture for enhanced gait feature extraction through combined spatial and temporal modeling. They later developed GaitGraph2 [20], featuring a multi-branch ResGCN [31] with branches for joints, bones, and motion. Similarly, Gao et al. [22] utilized GCNs to extract spatiotemporal dynamics from skeleton data sequences, addressing noise-related redundant information. Moreover, the studies in [23] explored the symmetry of human walking, such as the relationship between the left and right legs and hands, to capture dependencies in dynamic motion from skeleton data. However, these methods rely on a single biometric trait (i.e., skeleton data from one HPE algorithm), limiting their recognition accuracy, due to insufficient robustness in diverse real-world scenarios. This paper proposes a multi-biometric framework to improve the accuracy of skeleton-based gait recognition by utilizing multiple HPE algorithms.

## 3. Method

### 3.1. Overview

**Figure 2:**
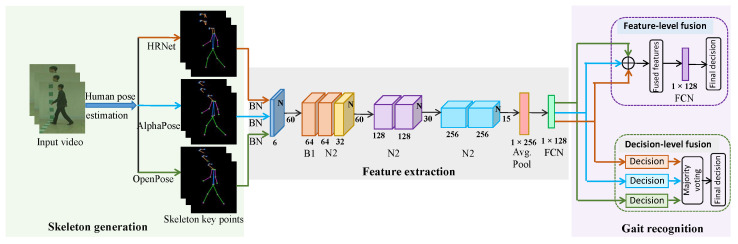
Overview of the proposed gait recognition framework. Given a raw RGB video sequence, the skeleton key points are extracted using a state-of-the-art pose estimation algorithm, and skeleton key points are preprocessed to generate input features. FC and ⊕ indicate the fully connected layer and element-wise addition, respectively.

This paper introduces a multi-biometric technique for person identification using gait features extracted from a single source video. We employed multiple HPE algorithms, OpenPose [15], AlphaPose [14], and HRNet [16], to obtain diverse skeleton key points. Subsequently, we utilized ResGCN [31] to extract spatiotemporal features from these skeleton samples. For the final prediction, we implemented both FLF and DLF. In FLF, the ResGCN model extracts features separately from each set of skeleton data points generated by the different HPE algorithms, and these features are then merged via pointwise addition for gait recognition. Conversely, DLF involves making individual predictions for each set of skeleton data points, and a final decision is reached using majority voting. An overview of the proposed framework is illustrated in Figure 2.

### 3.2. Human Pose Estimation

We employed three different HPE algorithms to extract skeleton data points from a single video source in our proposed multi-biometric person identification framework using gait features. We considered the state-of-the-art AlphaPose [15] and HRNet [16] as top-down algorithms, while OpenPose [14] was used as a bottom-up HPE algorithm.

Data preprocessing is a crucial step for skeleton-based gait recognition; following the preprocessing of the baseline model GaitGraph [19,20,31], we considered different data preprocessing techniques for the raw skeleton data points, for example, relative joint position (RJP), motion velocity (MV), and bone structure (BS) features. The RJP describes the positions of joints relative to a reference joint or relative to each other, which helps to capture the geometric structure of the human body. By contrast, BS provides the relative lengths and orientations of the bones formed by pairs of joints, providing a structural representation of the human body. At the same time, MV accounts for the speed and direction of joint movements over time, which is crucial for understanding the dynamic aspects of the gait. Please see the paper in [31] for more information.

### 3.3. Notation

The human skeleton can be represented as a graph G=(V,E), where $\left(V=v_{1},\ldots ,v_{N}\right)$ denotes a set of *N* nodes, each representing a joint in the skeleton, while *E* is a set of edges representing the connection among these joints. The connections are defined by an adjacency matrix $A\in R^{N\times N}$, where $A_{i,j}=1$ if there is an edge from node $v_{i}$ to node $v_{j}$, otherwise $A_{i,j}=0$. Here, the graph *G* is undirected, and the matrix *A* is symmetric. Each node $v_{n}$ in the graph contains three channels $v_{n}=\left(x_{n},y_{n},c_{n}\right)$, where *x* and *y* are the estimated coordinates of the joints and *c* is the confidence of the keypoint for that joint.

### 3.4. Graph Convolutions

In our network architecture, we employed graph convolutions. Given a skeleton data sequence *X* as input and graph structure *A*, the layer-wise update rule for graph convolutions applied to the features at time *t* can be expressed as

(1) $$X_{t}^{\left(l+1\right)}=\sigma \left({\tilde{D}}^{-\frac{1}{2}}\tilde{A}{\tilde{D}}^{-\frac{1}{2}}X_{t}^{\left(l\right)}W^{\left(t\right)}\right)$$

where $\tilde{A}=A+I$ represents the adjacency matrix of the skeleton graph with added self-loops, ensuring that each node retains its own feature as an identity feature. $\tilde{D}$ is a diagonal degree matrix corresponding to $\tilde{A}$, while σ(·) serves as an activation function, introducing non-linearity into the model. ${\tilde{D}}^{-\frac{1}{2}}\tilde{A}{\tilde{D}}^{-\frac{1}{2}}X_{t}^{\left(l\right)}$ are the aggregating spatial mean features from the messages passed from the immediate neighbors.

### 3.5. Feature Extraction and Fusion

We employed the ResGCN [31] architecture, initially developed for action recognition. This architecture, derived from the ST-GCN [36] block, sequentially executes spatial graph convolutions and temporal 2D convolutions, followed by batch normalization and ReLU activation. ResGCN [31] introduced a bottleneck structure inspired by ResNet [37], which adds two 1 × 1 convolutional layers before and after a convolution layer to reduce the number of feature channels and parameters.

**Feature-level fusion (FLF)** in multi-biometric techniques enhances the robustness and improves recognition accuracy by combining features through element-wise addition or concatenation. In our framework, we performed point-wise addition of features extracted from ResGCN applied to multiple skeleton data points generated using different HPE algorithms: OpenPose [14], AlphaPose [15], and HRNet [16]. Denoting the features extracted using ResGCN as $F_{op}$, $F_{al}$, and $F_{hr}$ for OpenPose, AlphaPose, and HRNet, respectively, FLF for gait recognition can be expressed as follows:

(2) $$\begin{array}{c} F_{com}=P_{add}\left(\left[F_{hr},F_{al},F_{op}\right]\right)\\ Y_{final}^{FLF}=GR\left(F_{com}\right) \end{array}$$

where $P_{add}\left(.\right)$ denotes the point-wise addition of the features $F_{hr}$, $F_{al}$, and $F_{op}$. And GR(.) performs gait recognition from the combined features (i.e., $Y_{com}$), and the final output from this method is $Y_{final}^{FLF}$; here, we considered a fully connected layer, followed by the Euclidean distance between the probe and gallery sample for final recognition.

**The decision-level fusion (DLF)** in multi-biometric techniques aggregates the outputs of multiple classifiers or models, combining their individual decisions to make a final prediction using methods such as majority voting or weighted voting. In our proposed framework, we utilize the output of the ResGCN classifier applied to features extracted from multiple HPE algorithms: OpenPose [14], AlphaPose [15], and HRNet [16]. The DLF can be expressed as follows:

(3) $$\begin{array}{c} Y_{hr}=GR\left(F_{hr}\right)\\ Y_{al}=GR\left(F_{al}\right)\\ Y_{op}=GR\left(F_{op}\right)\\ Y_{final}^{DLF}=MV\left(Y_{hr},Y_{al},Y_{op}\right) \end{array}$$

where GR(.) performs gait recognition using a fully connected layer, followed by the Euclidean distance between the probe and gallery sample for final recognition. Here, $Y_{hr}$, $Y_{al}$, and $Y_{op}$ denote the recognition outputs from using the skeleton data points generated by HRNet [16], AlphaPose [15], and OpenPose [14], respectively. The function MV(.) represents majority voting, which takes $Y_{hr}$, $Y_{al}$, and $Y_{op}$ as inputs and provides the final decision as $Y_{final}^{DLF}$.

### 3.6. Loss Function

We used supervised contrastive loss (SCL) [38] as the loss function. Unlike traditional contrastive losses like triplet or N-pairs loss, which typically consider a limited number of positive and negative samples, SCL takes into account all positive and negative samples within the batch. The compact nature of the skeleton data allows for the use of large batch sizes, ensuring each batch includes a positive pair. Elements with only negative pairs or no pairs are excluded from consideration. During training, the final feature $F_{com}$ is fed to the loss function (i.e., SCL) for feature-level fusion (FLF), while $F_{op}$, $F_{al}$, and $F_{hr}$ are fed separately for decision-level fusion (DLF).

## 4. **Experiments**

### 4.1. Dataset

The CASIA-B dataset [18] is one of the largest public gait datasets available for gait recognition. Unlike other datasets that provide silhouette images for public use, such as OU-MVLP [39] and OU-ISIR-bag [40], the CASIA-B dataset offers RGB color video sequence. This dataset includes 124 subjects (31 females and 93 males), each with ten distinct walking variations: six for normal walking (NM#01-06), two for walking while carrying bags (BG#01-02), and two for walking while wearing coats (CL#01-02). Each walking variation is captured from 11 distinct camera angles (0°, 18°, 36°, 54°, 72°, 90°, 108°, 126°, 144°, 162°, and 180°), resulting in 110 videos per subject. Specifically, each subject has 11 × (6 + 2 + 2) = 110 gait sequences, leading to a total of 110 × 124 = 13,640 gait sequences in the CASIA-B dataset. For our study, we used three different pre-trained pose estimation algorithms: HRNet [16], OpenPose [14], and AlphaPose [15] to extract skeleton data points.

### 4.2. Evaluation Settings

Following the experimental settings used in most research on gait recognition [20,23], we utilized widely used training and test split protocols on the CASIA-B [18] dataset for a fair comparison, as illustrated in Table 1. We segmented the dataset into training and test sets, where the training set comprised the first 74 subjects, and the test set included the remaining 50 subjects. The test set was further categorized into gallery and probe sets. The gallery set contained the initial four sequences (i.e., NM#01-04) of normal walking conditions. The probe set consisted of the final two sequences of normal walking (NM#01-02), two sequences of walking with a coat (CL#01-02), and two sequences of walking while carrying a bag (BG#01-02). The results were obtained across all viewing angles.

### 4.3. Implementation Details

We conducted all experiments on a single NVIDIA 3090 GPU running the Linux operating system. The experiments were implemented using Python 3.9.2 and PyTorch 1.10.0. We utilized the Adam optimizer [41] with a 1-cycle learning rate policy and a weight decay penalty of 1 $\times \;10^{-5}$. To generate the skeleton input, we maintained a consistent sequence length of T = 60 frames across all three pose estimators for the CASIA-B dataset. Both the batch size and the embedding layer size were configured to 128. During the initial phase of training, the maximum learning rate was set to 1 $\times \;10^{-2}$ for 300 epochs. In the subsequent phase, the maximum learning rate was adjusted to 1 $\times \;10^{-5}$ for an additional 100 epochs.

### 4.4. Comparison with State-of-the-Art Methods

**Evaluation on the CASIA-B Dataset.** We evaluated our proposed multi-biometric framework on the CASIA-B dataset [18] and compared its performance against several state-of-the-art model-based approaches. The Rank-1 accuracy results on CASIA-B are presented in Table 2. Compared to PTSN [12], PoseGait [12], Siamese [42], GaitGraph [19], GaitGraph2 [20], ResGait [22], SDHF-GCN [23], and LuGAN-HGC [24], our proposed multi-biometric method achieved superior accuracies. Specifically, our framework a demonstrated state-of-the-art rank-1 accuracy across 11 probe views (excluding identical-view cases) for normal walking (NM), walking with a carried object (BG), and walking with clothing variation (CL) conditions. In particular, the DLF method achieved rank-1 accuracies of 91.8%, 79.0%, and 68.9% for NM, BG, and CL conditions, respectively, while the FLF method achieved 93.3%, 81.3%, and 72.4% for the same conditions. These results indicate that our DLF method outperformed the PTSN [12] by substantial margins of 44.4%, 50.7%, and 51.3% for the NM, BG, and CL conditions, respectively, while FLF surpassed the PTSN by margins of 45.9%, 53.0%, and 54.8%.

We can observe that our proposed multi-biometric techniques, DLF and FLF, surpassed the accuracies of the baseline model GaitGraph2 [20] by a large margin. Specifically, DLF achieved improvements of 9.8%, 5.8%, and 5.3% for the NM, BG, and CL walking conditions, respectively, while FLF achieved improvements of 11.3%, 8.1%, and 8.8%. Furthermore, our proposed FLF obtained the best or second-best accuracies for each probe viewing angle for all the gallery view angles, excluding the identical view angles.

### 4.5. Evaluation of Proposed Multi-Biometric Technique

The proposed multi-biometric framework in this study included several HPE algorithms to extract skeleton data points for gait recognition, including OpenPose [14], AlphaPose [15], and HRNet [16]. We conducted an experiment using each of the extracted skeleton data samples separately to highlight the effectiveness of our proposed multi-biometric technique. Moreover, we compared the accuracy of the proposed framework with that of the baseline model, i.e., GaitGraph2 [20], to demonstrate the superiority of our proposed framework.

**Figure 3:**
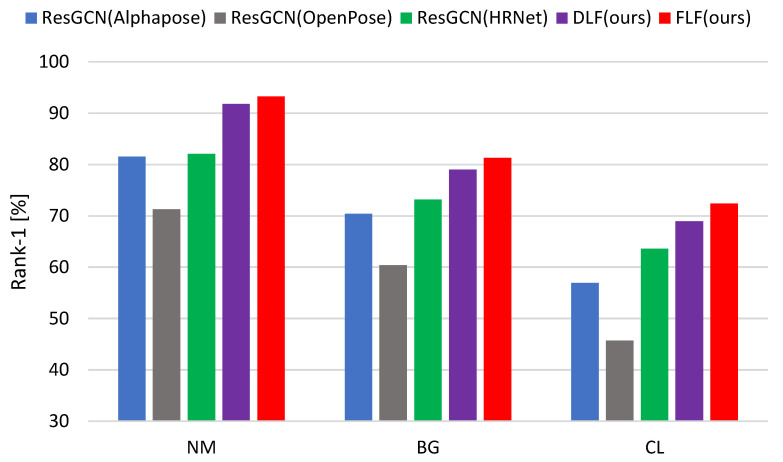
The average recognition rates of DLF and FLF along with the baseline algorithms on the CASIA-B dataset for normal walking (NM), carrying bags (BG), and wearing coats (CL) sequences.

**Comparison with separate HPE algorithms**. Here, we present the overall comparison among the various HPE algorithms used to extract skeleton data points for gait recognition. The results of the skeleton data points extracted from the CASIA-B dataset are reported in Table 3 and Figure 3. The multi-biometric techniques, FLF and DLF, consistently achieved higher accuracy across all covariate conditions, NM, BG, and CL, compared to the individual HPE algorithms. For example, FLF/DLF obtained the rank-1 accuracy of 93.3%/91.8%, 81.3%/79.0%, and 72.4%/68.9%, respectively, for the NM, BG, and CL conditions. This result indicated that our FLF/DLF surpassed the 22.0%/20.5%, 20.9%/18.6%, 26.7%/23.2% of the OpenPose algorithm, and the 11.2%/9.7%, 8.1%/5.8%, and 8.8%/5.3% from HRNet. Our proposed multi-biometric technique incorporated multiple HPE algorithms to extract diverse skeleton data points and demonstrated superior performance, due to its ability to integrate diverse information effectively. Regarding FLF, our proposed multi-biometric framework performs better in gait recognition by combining features from multiple HPE algorithms, OpenPose [14], AlphaPose [15], and HRNet [16], at the feature extraction level. This method leverages the strengths and mitigates the weaknesses of individual feature sets, resulting in a more comprehensive and discriminative representation.

By contrast, DLF makes the final decision by aggregating the individual decisions from the baseline method, ResGCN [31], applied separately to the skeleton data points extracted using different HPE algorithms. By using majority voting, DLF capitalizes on the complementary nature of the various algorithms and reduces the impact of inaccuracies or imperfect skeleton generation by a single HPE algorithm. For example, while HRNet [16] performs better with low-resolution images, other algorithms may perform better under different conditions. DLF provides a more reliable final output by aggregating their outputs as majority voting, leading to an improved overall gait recognition accuracy. Moreover, we see that among the HPE algorithms, HRNet [16] obtained a comparatively higher accuracy than OpenPose [14] and AlphaPose [15].

**Figure 4:**
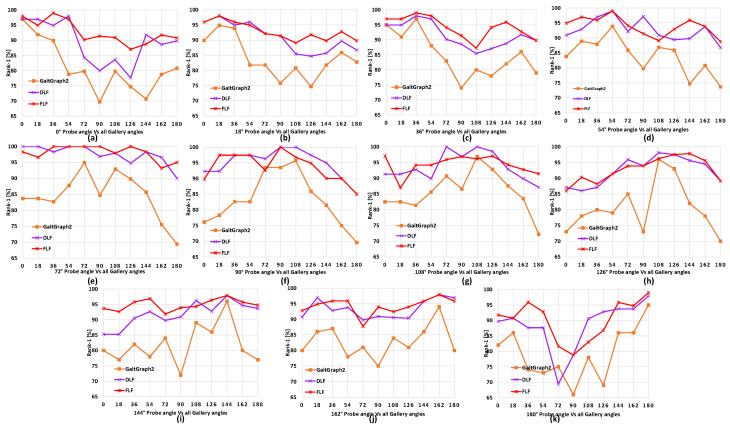
Comparison of the proposed DLF and FLF with baseline pose estimator algorithms results on the CASIA-B dataset. This consists of 11 subgraphs, each denoting a probe view angle against all gallery view angles for normal walking (NM) sequences. Best viewed in color.

**Figure 5:**
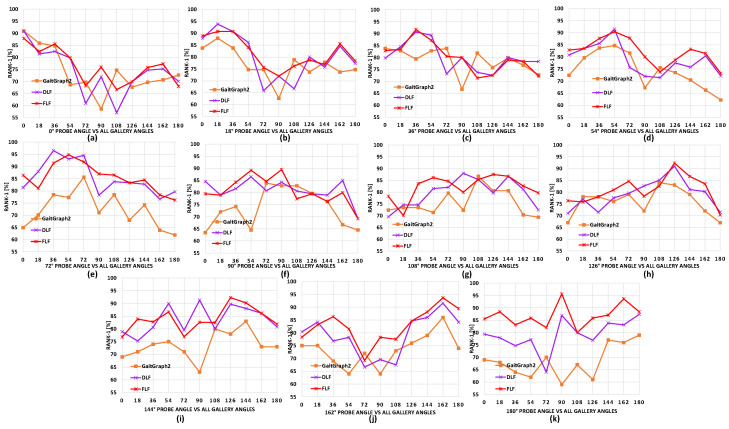
Comparison of the proposed DLF and FLF with baseline pose estimator algorithms results on the CASIA-B dataset. This consists of 11 subgraphs, each denoting a probe view angle against all gallery view angles for carrying bags (BG) sequences. Best viewed in color.

**Figure 6:**
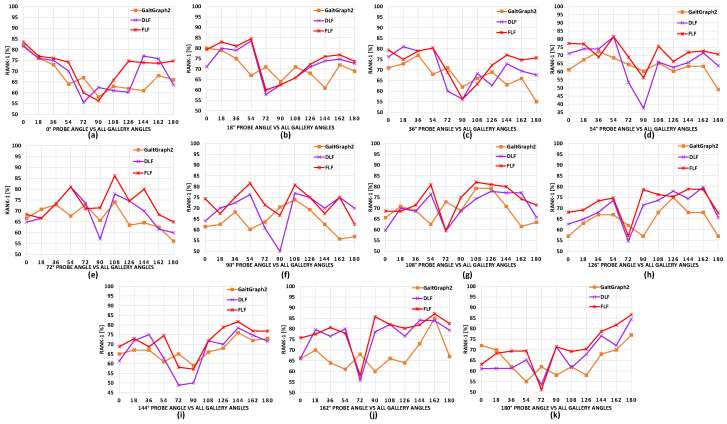
Comparison of the proposed DLF and FLF with baseline pose estimator algorithms results on the CASIA-B dataset. This consists of 11 subgraphs, each denoting a probe view angle against all gallery view angles for wearing coats (CL) sequences. Best viewed in color.

**Comparison with baseline model**. Here, we present overall comparisons with the baseline model GaitGraph2 [20], with the skeleton data points and using HRNet [16] according to each separate probe angle for all gallery view angles. The results on CASIA-B are shown in Figure 4, Figure 5 and Figure 6. We can observe that for most of the viewing angles, the recognition accuracy was comparatively higher for DLF and FLF for the NM, BG, and CL conditions, as shown in Figure 4.

**Comparison between FLF and DLF**. Here, we present a comparison between the multi-biometric techniques FLF and DLF. The results are shown in Table 2 and Table 3 along with Figure 4, Figure 5 and Figure 6. We can observe that FLF consistently outperformed DLF. This was likely due to the lower quality of skeleton data points extracted by OpenPose [14] and AlphaPose [15], as shown in Figure 1, which led to a reduced recognition accuracy compared to HRNet. Specifically, HRNet produced accurate skeletons, while AlphaPose and OpenPose exhibited limitations in keypoint detection during self-occlusions, with OpenPose occasionally losing body segments during leg swings and self-occluded poses. Consequently, when the ResGCN model was applied to the skeleton data from OpenPose and AlphaPose, these inaccuracies led to incorrect recognitions, resulting in more false recognitions by DLF due to its majority voting mechanism. In contrast, FLF combined features through pointwise addition, which preserved their discriminative capability and enhanced the overall recognition performance.

## 5. Conclusions

In this paper, we introduced a multi-biometric technique for skeleton-based gait recognition. Our approach utilizes multiple top-down and bottom-up human pose estimation (HPE) algorithms to extract skeleton data points, ensuring a comprehensive capture of gait features. We proposed using both feature-level fusion (FLF) and decision-level fusion (DLF) to enhance the recognition accuracy. FLF combines features through pointwise addition, leveraging the complementary strengths of different HPE algorithms, while DLF aggregates decisions from individual models using a majority voting mechanism. We validated the effectiveness of our framework on the widely used CASIA-B gait database. The results demonstrated that our proposed multi-biometric technique significantly improved the recognition performance, achieving state-of-the-art accuracy for model-based gait recognition. This study underscores the potential of integrating multiple HPE algorithms and fusion techniques to enhance the robustness and accuracy of gait recognition systems.

While this study achieved state-of-the-art accuracy on the small-scale CASIA-B dataset, it has limitations and opportunities for improvement. The CASIA-B dataset, with 124 subjects across 11 distinct view angles, includes limited covariates, such as controlled conditions for carrying a single small bag and only two clothing variations, which may not fully capture the diversity needed for real-world applications. Future research would benefit from large-scale datasets with a broader range of covariates, such as occlusions, diverse clothing types, and realistic camera view angles, to enhance the robustness and generalizability. Additionally, incorporating 3D instrumented gait analysis as a gold standard could improve validation, while testing on real-time samples, such as security camera footage, would offer insights into practical performance. These steps would support a broader applicability in real-world scenarios.

## Figures and Tables

**Table 1:** Experimental setting using the CASIA-B dataset.

Training	Test	
	Gallery Set	Probe Set
ID: 001-074	ID: 075-124	ID: 075-124
Seqs.: NM#1-6, BG#1-2, CL#1-2	Seqs.: NM#1-4	Seqs.: NM#5-6, BG#1-2, CL#1-2

**Table 2:** Rank-1 accuracy (%) on the CASIA-B dataset for 11 probe views, excluding identical-view cases, compared with other skeleton-based methods. Here, FLF and DLF denote feature-level fusion and decision-level fusion, respectively. Values in bold and italic indicate the best and second-best benchmarks, respectively.

Gallery nm#1-4		0–180°											Mean
Probe	Methods	0°	18°	36°	54°	72°	90°	108°	126°	144°	162°	180°	
NM#5-6	PTSN [12]	34.5	45.6	49.6	51.3	52.7	52.3	53.0	50.8	52.2	48.3	31.4	47.4
	PoseGait [12]	55.3	69.6	73.9	75.0	68.0	68.2	71.1	72.9	76.1	70.4	55.4	68.7
	Siamese [42]	72.4	81.2	85.6	80.4	79.4	85.0	81.0	77.6	82.5	79.1	80.2	80.4
	GaitGraph [19]	85.3	88.5	91.0	92.5	87.2	86.5	88.4	89.2	87.9	85.9	81.9	87.7
	GaitGraph2 [20]	78.5	82.9	85.8	85.6	83.1	81.5	84.3	83.2	84.2	81.6	71.8	82.0
	ResGait [22]	85.2	88.4	92.8	90.3	**93.2**	*90.5*	91.3	89.6	88.6	89.7	85.8	89.6
	SDFH-GCN [23]	77.3	82.8	85.1	86.0	85.5	85.4	83.7	81.5	80.5	83.9	77.6	82.7
	LuGAN-HGC [24]	89.3	88.1	89.0	89.9	87.4	88.7	87.4	88.8	88.8	87.0	87.0	88.3
	DLF (ours)	*91.8*	*92.7*	*93.3*	*94.4*	90.0	*90.5*	**91.9**	*90.5*	*92.8*	*92.3*	89.5	*91.8*
	FLF (ours)	**93.7**	**93.8**	**95.8**	95.8	*91.4*	**92.3**	*91.7*	**93.5**	**94.3**	**93.3**	91.0	**93.3**
BG#1-2	PTSN [12]	22.4	29.8	29.6	29.2	32.5	31.5	32.1	31.0	27.3	28.1	18.2	28.3
	PoseGait [12]	35.3	47.2	52.4	46.9	45.5	43.9	46.1	48.1	49.4	43.6	31.1	44.5
	Siamese [42]	62.5	68.7	69.4	64.8	62.8	67.2	68.3	65.7	60.7	64.1	60.3	65.0
	GaitGraph [19]	75.8	76.7	75.9	76.1	71.4	73.9	*78.0*	74.7	75.4	75.4	69.2	74.8
	GaitGraph2 [20]	69.9	75.9	78.1	79.3	71.4	71.7	*74.3*	76.2	73.2	73.4	61.7	73.2
	ResGait [22]	73.5	78.2	79.6	83.3	**82.4**	78.5	**81.7**	*81.1*	78.4	80.3	74.2	*79.2*
	SDFH-GCN [23]	67.5	73.9	73.2	74.3	68.5	68.5	70.5	69.0	62.2	68.7	60.1	68.8
	LuGAN-HGC [24]	79.4	79.5	81.6	82.4	78.1	76.2	78.7	82.0	81.6	83.0	73.6	79.7
	DLF (ours)	*79.4*	*80.5*	*81.5*	*83.9*	72.8	*79.3*	74.6	79.4	*80.6*	*81.1*	*75.6*	79.0
	FLF (ours)	**81.6**	**81.1**	**85.3**	**85.6**	*79.4*	**81.0**	77.5	**81.3**	**82.4**	**82.7**	**75.9**	**81.3**
CL#1-2	PTSN [12]	14.2	17.1	17.6	19.3	19.5	20.0	20.1	17.3	16.5	18.1	14.0	17.6
	PoseGait [12]	24.3	29.7	41.3	38.8	38.2	38.5	41.6	44.9	42.2	33.4	22.5	36.0
	Siamese [42]	57.8	63.2	68.3	64.1	*66.0*	64.8	67.7	60.2	66.0	68.3	60.3	64.2
	GaitGraph [19]	*69.6*	66.1	68.8	67.2	64.5	62.0	69.5	65.6	65.7	66.1	64.3	66.3
	GaitGraph2 [20]	*57.1*	61.1	68.9	66.0	67.8	65.4	68.1	67.2	63.7	63.6	50.4	63.6
	resgait [22]	64.2	68.3	**74.6**	*75.8*	**71.6**	**72.4**	69.1	*70.8*	67.6	70.5	67.1	*70.2*
	SDHF-GCN [23]	63.4	65.4	66.7	64.8	63.0	66.2	69.1	63.3	61.1	65.9	60.7	64.5
	LuGAN-HGC [24]	72.8	72.3	69.4	75.2	77.0	79.6	80.5	78.1	76.3	74.9	72.8	75.4
	DLF (ours)	65.9	*71.5*	72.3	74.9	56.0	61.6	*70.5*	69.8	*74.2*	*73.2*	*68.0*	68.9
	FLF (ours)	**72.3**	**72.0**	*73.8*	**77.9**	61.3	*67.1*	**73.7**	**74.5**	**76.6**	**75.3**	**72.1**	**72.4**

**Table 3:** Comparison table of the average rank-1 accuracy (%) with baseline algorithms using ResGCN [31] on the CASIA-B dataset for normal walking (NM), carrying bags (BG), and wearing coats (CL) sequences.

Methods	Pose Estimation Algorithms			Rank-1 [%]		
	OpenPose	Alphapose	HRNet	NM	BG	CL
ResGCN	✓			71.3	60.4	45.7
ResGCN		✓		81.5	70.4	56.9
ResGCN			✓	82.1	73.2	63.6
DLF (ours)	✓	✓	✓	91.8	79.0	68.9
FLF (ours)	✓	✓	✓	93.3	81.3	72.4

## Data Availability

The data and source code will be made available upon request.
